# Morphometric assessment of spleen dimensions and its determinants among individuals living in Arba Minch town, southern Ethiopia

**DOI:** 10.1186/s12880-021-00719-9

**Published:** 2021-12-04

**Authors:** Solomon Demissie, Prasad Mergu, Tadiwos Hailu, Getachew Abebe, Mengistu Warsa, Teshale Fikadu

**Affiliations:** 1 Department of Clinical Anatomy, College of Medicine and Health Science, Arba Minch University, Arba Minch, Ethiopia; 2 Department of Anatomy, MNR Medical College and Hospital, Sagerddy, Telangana India; 3 Department of Internal Medicine, College of Medicine and Health Science, Arba Minch University, Arba Minch, Ethiopia; 4 Department of Medicine, Arba Minch General Hospital, Arba Minch, Ethiopia; 5 Department of Public Health and Epidemiology, College of Medicine and Health Science, Arba Minch University, Arba Minch, Ethiopia

**Keywords:** Morphometry, Spleen dimensions, Arba Minch

## Abstract

**Introduction:**

The spleen is a vital lymphoid soft organ that demands constant attention from the clinical point of view. It is a multi-dimensional organ that enlarges in its all dimensions during some disease condition. The detection of the spleen by palpation is not an indicator of an enlarged spleen because normal spleen may be palpable. Therefore, this study aimed to assess the morphometry of spleen dimensions and its determinants among individuals living in Arba Minch town by sonographic examinations.

**Methods and materials:**

Community-based cross-sectional study was conducted in Arba Minch town from February 1 to March 30, 2020. Seven hundred and eight study participants were selected using a multi-stage systematic random sampling technique. Data were checked for completeness, edited, coded and entered into Epi-Data version 3.1 and exported to STATA software version 16 for analysis.

**Result:**

The mean splenic length, width, thickness and volume were 10.24 cm, 4.79 cm, 3.93 cm, and 109.34 cm^3^, respectively. The mean spleen length, width, thickness and volumes among males were 10.64 cm, 4.92 cm, 4.05 cm and 119.81 cm^3^ and among females were 9.75 cm, 4.63 cm, 3.78 cm and 96.50 cm^3^ respectively. As age increased by one year the mean spleen length, width, thickness and volume was decreased by 0.032 cm, 0.018 cm 0.004 cm and 0.012 cm respectively. As height increased by 1 cm the mean spleen width and volume were increased by 0.096 cm and 0.052 cm respectively. As we go from male to female the mean spleen length decreased by 0.294 cm.

**Conclusion:**

The spleen dimensions were higher in males than females. Splenic length was determined by age & sex, the spleen width was determined by age & height, the spleen volume was determined by age & height and the spleen thickness was determined by age.

## Introduction

The spleen is the largest lymphoid organ located in the left hypochondrium between the fundus of the stomach and the diaphragm where it is entirely covered by the inferior thoracic rib cage [1]. It extends from the 9th–11th ribs on the left side with its long axis runs parallel to the 10th rib [2, 3]. Its shape is ovoid-like with a convex outer diaphragmatic surface and an indented inner visceral surface related to the stomach, left kidney, pancreatic tail, left suprarenal gland and left colic flexure [4]. The apex lies in line with the spine of the 10th thoracic vertebra about 4 cm from the midline and the base does not descend beyond the midaxillary line [4].

The spleen is an encapsulated intraperitonial organ entirely covered with peritoneum except for its hilum where the splenic branches of the splenic artery and vein enter and leave [5]. It is supported by a phrenico-colic ligament from the bottom. It is anchored to the stomach by gastro-splenic ligament and to the left kidney by a lien renal ligament [6].

In diseases condition spleen enlarges at different rates in its all dimensions. A variety of diseases condition alters spleen dimensions, where splenomegaly and its consequence becomes a primary clinical concern in developing countries [7]. It is commonly seen in about 63% of patients with Pulmonary arterial hypertension [8], Infectious Mononucleosis [9], malaria [10], lymphoma [11], kala-azar [12], typhoid fever [13], liver disease (hepatitis and cirrhosis) [14], haematological diseases, metabolism diseases and cancer [15]. The altered splenic dimensions and structure during these diseases result in asymptomatic enlargement and complications such as hematoma formation, rupture, hypersplenism, ectopic spleen, and torsion that affect other adjacent organs [16].

Splenic atrophy is also another common problem seen in diseases like sickle cell anaemia where the progressive atrophy as a result of repeated attacks of vaso-occlusion and infarction caused by these diseases leads to auto splenectomy [17].

The average dimensions of the spleen are 12.5 cm, 7.5 cm and 2.5 cm in length, width and thickness respectively and 150–200 g in weight, but its dimensions vary considerably [18]. The literature revealed that spleen dimensions are affected by geographical differences, races, nutritional status and anthropometric measurements [19–21].

In clinical practice, palpation is commonly used to detect spleen enlargement. However, detection of spleen by palpation is not reliable or (not accurate) and might lead to misdiagnosis [2, 22, 23]. Palpable spleen is not pathological for some individuals [24]. Moreover, enlarged spleen below 40% increment might not be detected by palpation [25].

Although, literatures indicates radiological imaging modalities like ultrasound can detect the extent, complications, and classify the severity of cases, yet enough attention is not given in clinical practice [26]. On sonographic examinations, the spleen is crescent-shaped with smooth and convex outer margin and irregularly indented inner margin. Its echotexture is homogeneous isoechogenic and is the same or very similar to that of the healthy liver tissue, and similar to or slightly lower than the echotexture of the renal cortex, or markedly higher than that of the renal medulla [27].

Even though there are several studies in which the spleen dimensions have been analysed in many countries, there is still a lack of adequate information in African populations including Ethiopia. Therefore, the study was aimed to assess the morphometry of spleen dimensions and to determine its variations with sex, age, height, weight, body mass index (BMI), and body surface area (BSA) among individuals living in Arba Minch town.

## Methods and materials

The community-based cross-sectional study was conducted from February 1 to March 30, 2020, in Arba Minch town, Southern Nations, Nationalities and Peoples Region (SNNP) which is located at an altitude of 1285 m above sea level and 437 km south of Addis Ababa (capital city of Ethiopia).

The data was collected using data collection checklist. The data collection checklist was developed in English after reviewing different literature and the face validity was assessed by a public health specialist, radiologist and anatomist. The checklist was composed of socio-demographic factors (age and sex); anthropometric measurements (height, weight, BSA and BMI) and spleen dimensions (length, width, thickness and volume).

**Fig. 1:**
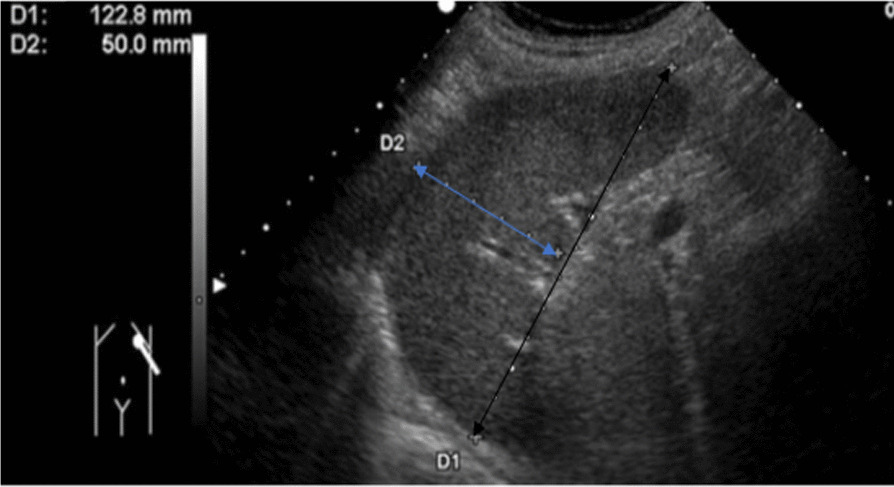
Spleen length (black arrow) and width (blue arrow) measured on longitudinal ultrasound scan

**Fig. 2:**
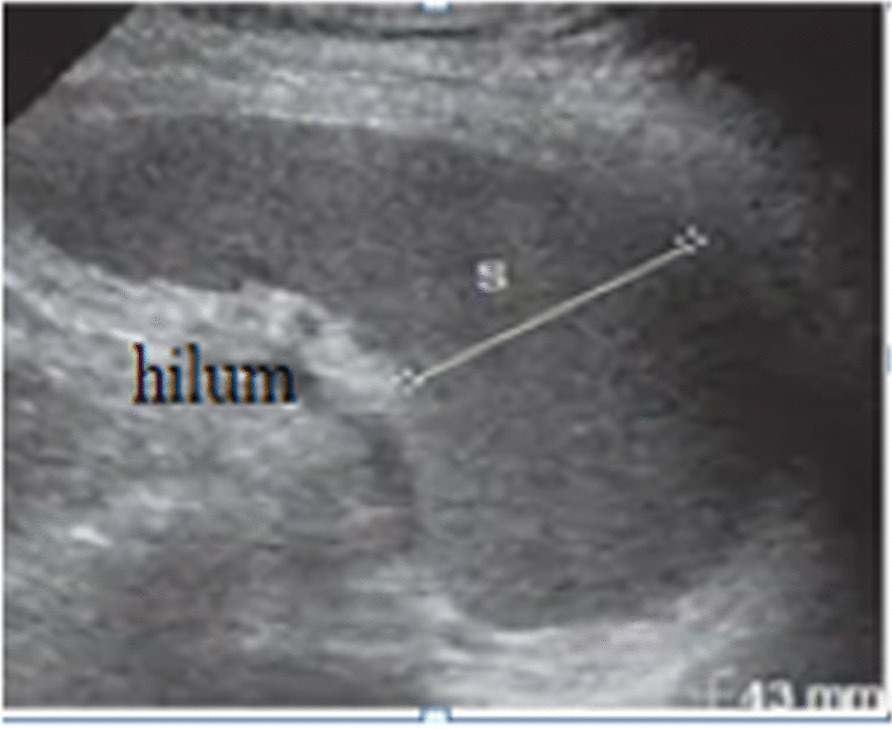
Spleen thickness measured on transverse ultrasound scan (white line)

Seven hundred and eight individuals (390 males and 318 females) fulfilling inclusion criteria were selected by multi-stage systematic random sampling technique. During the procedures, individuals were first selected randomly at household levels by distributing questionnaires which include the inclusion criteria. Then the individuals fulfilling the inclusion criteria were sent to the nearest health center or selected private clinics for clinical and sonographic examination. During examinations, history and physical examination of individuals were taken before sonographic examination by physician. Then sonographic examinations were performed by two radiology technologists using portable and stationary ultrasound machines equipped with 3.5 MHz convex probes. During sonographic examination, the subjects were examined in the supine or right oblique positions. After assessment of overall abdominal sonographic examinations, the spleen dimensions measurements were performed for those with no case findings. The measurement was performed during suspended respiration. The splenic length was measured in a longitudinal plane between the dome of the spleen and the splenic tip [3, 28] (Fig. 1). The splenic width was also measured in a longitudinal plane perpendicular to the length between the medial and lateral borders of the spleen [20] (Fig. 1). The splenic thickness was measured on the transverse plane from the posterior margin to the anterior margin [18] (Fig. 2). The volume was calculated using the ellipsoid formula during the analysis [29, 30]. The spleen dimensions were measured three times and recorded on the checklist then the average value was taken during analysis [31]. Finally, the baselines data including age and sex were recorded for all participants. The height and weight were measured with the stadiometer and weighing machine for all participants respectively. BSA and BMI were calculated during the analysis.

### Inclusion and exclusion criteria

#### Inclusion criteria

All apparently healthy individuals and age greater than 13 years [32].

#### Exclusion criteria

- Individuals with a recurrent clinical history of malaria
- Recurrent history of typhoid fever
- Individuals with a history of infections (infectious mononucleosis, kala-azar, endocarditis, sarcoidosis, toxoplasmosis).
- Recent history of upper abdominal surgery
- Individuals with any case finding on sonographic examinations (cirrhosis, melanoma, lymphoma, metastasis, any cystic or solid massive lesions)
- Individuals with Diabetic Mellitus (DM) cases.
- Hypertensive individuals
- Individuals with a history of heart disease
- Individuals with a recent abdominal traumatic condition (within the previous 6 months)
- Pregnant women
- History of sickle cell anaemia
- Very old age

### Operational definitions

Apparently healthy individuals: is defined as the absence of disease based on clinical signs and symptoms of individuals assessed by history, physical evaluation and sonographic examinations [33, 34].

### Data analysis

Data was checked for completeness, edited, coded and entered into Epi-Data version 3.1 and exported to STATA software version 16 for analysis. A descriptive summary was used to present the result. Independent- Samples t-test was conducted to compare the mean spleen dimensions among sex and one-way ANOVA was conducted to compare the mean spleen dimensions among age groups. T and F statistics were calculated and *p* < 0.05 was taken as statistically significant. Linear regression model were fitted to identify determinants of spleen dimension. Square root and logarithmic transformation were used for thickness and volume respectively to full fill the assumption of linear regression.

## Result

A total of 708 participants were included in the current study with a 100% response rate. The mean splenic length, width, thickness, and volume were 10.24 ± 1.45 cm, 4.79 ± 0.99 cm, 3.93 ± 1.05 cm, and 109.34 ± 61.68 cm^3^, respectively (Table 1).

**Table 1:** Average spleen dimensions of study participants living in Arba Minch town, 2020

Variable	n	Mean	SD	[95%_CI]	
Length	708	10.24	1.45	10.14	10.35
Width	708	4.79	0.99	4.72	4.86
Thickness	708	3.93	1.05	3.85	4.01
Volume	708	109.32	61.69	104.77	113.87

### Socio-demography

The mean age of the study participant was 32.28 ± 13.17 years. More than one half of the respondent were males (55.08%) and more than one third (39.3%) were between the age group of 21–30 years. Majority of participants (82.49%) had normal BMI (Table 2).

**Table 2:** Socio demographic characteristics of study participant living in Arba Minch town, 2020

Variable	Frequency	Percent
Sex of the respondent		
Male	390	55.08
Female	318	44.92
Age of the respondent		
11–20	130	18.36
21–30	278	39.27
31–40	154	21.75
41–50	66	9.32
> 50	80	11.30
BMI of the respondent		
Under weight	40	5.65
Normal weight	584	82.49
Over weight	84	11.86

### Anthropometric measurements

The mean height, weight, BMI, and BSA were 167.56 ± 6.69 cm, 61.75 ± 8.23 kg, 21.98 ± 2.53, and 1.44 ± 0.23 respectively (Table 3).

**Table 3:** Anthropometric measurement of the study participants living in Arba Minch town, 2020

Variable	N	Mean	SD	Min	Max
Height	708	167.56	6.69	150	188
Weight	708	61.75	8.23	44	89
BMI	708	21.98	2.53	14.82	30.675
BSA	708	1.44	0.23	0.95	2.213

### Comparison of spleen dimensions by sex

The mean spleen length, width, thickness and volume among males were 10.64 cm, 4.92 cm, and 4.05 cm and among females were 119.77 cm^3^ and 9.75 cm, 4.63 cm, 3.78 cm and 96.50 cm^3^ respectively. Significant differences were observed in spleen dimensions among males and females (Table 4).

**Table 4:** Comparison of spleen dimensions by sex among individuals living in Arba Minch town, 2020

	Male(1)	Female(2)	Mean1	Mean2	Difference	SE	t value	*p* value
Length	390	318	10.64	9.75	0.89	.104	8.55	0.01
Width	390	318	4.92	4.63	0.28	.074	3.75	0.01
Thickness	390	318	4.05	3.78	0.27	.079	3.45	0.01
Volume	390	318	119.77	96.50	23.27	4.581	5.1	0.01

### Comparisons of spleen dimensions with different age groups

Significant variations were observed in all spleen dimensions among age categories of study participants. Bonferroni test for multiple comparisons found that the change in spleen length was significantly higher for 31–40 years compared to 11–20 years old (0.62, *p* = 0.002), 11–20 year compared to > 50 years (0.87, *p* = 0.001), 21–30 years compared to 41–50 years (0.66, *p* = 0.006), 21–30 years compared to > 50 years (1.02, *p* = 0.001), and 31–40 years compared to 41–50 years (1.04, *p* = 0.001). The change in spleen width was significantly higher for 11–20 years compared to > 50 years old (0.55, *p* = 0.001), 21–30 year compared to > 50 years (0.51, *p* = 0.001), and 31–40 years compared to > 50 years (0.66, *p* = 0.006). The change in spleen thickness was significantly higher for 31–40 years compared to 41–50 years old (0.47, *p* = 0.022), 11–20 year compared to > 50 years (0.44, *p* = 0.030), and 31–40 years compared to > 50 years (0.64, *p* = 0.001). The change in spleen volume was significantly higher for 31–40 years compared to 41–50 years old (29.82, *p* = 0.009), 21–30 year compared to > 50 years (23.34, *p* = 0.025), and 31–40 years compared to > 50 years (39.75, *p* = 0.001) (Table 5).

**Table 5:** Comparison of spleen dimensions with age groups among the study participants living in Arba Minch town, 2020

	Age	Mean	SD	F	*p*-value	Bonferroni comparison			
						11–20	21–30	31–40	41–50
Length	11–20	10.14	1.21	16.53	0.001				
	21–30	10.38	1.31			0.24			
	31–40	10.76	1.49			0.62	0.38		
	41–50	9.72	1.57			0.42	0.66	1.04	
	> 50	9.36	1.56			0.78	1.02	1.40	0.36
Width	11–20	4.90	0.90	6.43	0.001				
	21–30	4.85	0.92			0.05			
	31–40	4.91	1.08			0.01	0.06		
	41–50	4.54	1.04			0.36	0.32	0.37	
	> 50	4.35	1.08			0.55	0.51	0.56	0.19
Thickness	11–20	4.00	0.87	5.98	0.001				
	21–30	3.90	1.00			0.09			
	31–40	4.20	1.21			0.20	0.30		
	41–50	3.73	1.09			0.27	0.17	0.47	
	> 50	3.56	1.05			0.44	0.35	0.64	0.17
Volume	11–20	109.61	51.77	6.56	0.001				
	21–30	109.65	54.88			0.04			
	31–40	126.05	71.30			16.44	16.40		
	41–50	96.23	69.10			13.38	13.42	29.82	
	> 50	86.30	64.18			23.30	23.34	39.75	9.93

### Determinants of spleen dimensions

The splenic length was determined by age and sex, as age increased by one year the mean spleen length was decreased by 0.032 cm. when we compare males and females spleen length the mean spleen length decreased by 0.294 cm in case of female. The splenic width was determined by age and height. As age increased by one year the mean spleen width was decreased by 0.018 cm. As height increased by 1 cm the mean spleen width was increased by 0.096 cm. The splenic thickness was determined by age only. As age increased by one year the mean spleen width was decreased by 0.004 cm. The splenic volume was determined by age and height. As age increased by one year the mean spleen volume was decreased by 0.012 cm. Also as height increased by 1 cm the mean spleen volume was increased by 0.052 cm (Table 6).

**Table 6:** Determinants of spleen dimensions among individuals living in Arba Minch town, 2020

Length	Coef	SE	t-value	*p*-value	[95% Conf	Interval]	Sig
Age	− 0.032	0.004	− 9.15	0.000	− 0.039	− 0.025	***
Male	Ref						
Female	− 0.294	0.095	− 3.10	0.002	− 0.481	− 0.108	***
Height	0.067	0.055	1.21	0.225	− 0.041	0.176	
Weight	0.392	0.324	1.21	0.227	− 0.245	1.028	
BMI	− 0.294	0.369	− 0.80	0.425	− 1.019	0.43	
BSA	− 9.521	8.82	− 1.08	0.281	− 26.838	7.796	
Width							
Age	− 0.018	0.003	− 6.54	0.000	− 0.023	− 0.012	***
Male	0						
Female	0.096	0.074	1.30	0.196	− 0.049	0.241	
Height	0.094	0.043	2.18	0.029	0.009	0.178	**
Weight	0.217	0.252	0.86	0.388	− 0.277	0.711	
BMI	− 0.06	0.286	− 0.21	0.833	− 0.622	0.502	
BSA	− 7.17	6.847	− 1.05	0.295	− 20.614	6.273	
Thickness							
Age	− 0.004	0.001	− 5.28	0.000	− 0.005	− 0.002	***
Male	0						
Female	0.02	0.02	0.98	0.33	− 0.02	0.059	
Height	0.017	0.012	1.44	0.15	− 0.006	0.04	
Weight	0.096	0.069	1.40	0.162	− 0.039	0.231	
BMI	− 0.063	0.078	− 0.81	0.42	− 0.216	0.09	
BSA	− 2.786	1.866	− 1.49	0.136	− 6.449	0.877	
Volume							
Age	− 0.012	0.001	− 8.30	0	− 0.015	− 0.009	***
Male	0						
Female	0.017	0.039	0.45	0.652	− 0.059	0.094	
Height	0.052	0.023	2.30	0.022	0.008	0.096	**
Weight	0.216	0.132	1.64	0.102	− 0.043	0.476	
BMI	− 0.117	0.15	− 0.78	0.436	− 0.413	0.178	
BSA	− 6.566	3.596	− 1.83	0.068	− 13.626	0.495	

^***^p < .01, **p < .05

## Discussion

This study describes the morphometry of spleen dimensions; compare the presence of a significant difference between sex and age. The study also assesses the determinant factors of spleen dimensions. The sonography assessment of spleen dimensions provides essential inputs for clinicians in daily clinical practice for the proper diagnosis of splenomegaly [21, 35, 36]. This study provides estimates of spleen to help radiologist for the diagnosis of diseases related to splenomegaly and atrophy also used for haematologist and immunologist for the diagnosis of various gastrointestinal and haematological diseases in addition to forensic studies [37–39].

In this study the mean spleen length was 10.24 cm which is consistent with studies conducted in Russia and Kashmir [40, 41]. But, less than from studies conducted in Turkey, Bangladesh, Jordan and North India [29, 42–44]. It is greater than from studies conducted in Nepal, Nigeria, Sudan and Northern Ethiopia [18, 19, 45, 46].

The mean spleen width was 4.79 cm which is less than studies conducted in Bangladesh, Nepal, Kashmir, North India and Nigeria [41, 42, 44–47]. It is greater than from studies conducted in Sudan and Northern Ethiopia [18, 19].

The mean spleen thickness in the current study was 3.93 cm which is consistent with the study conducted in Northern Ethiopia [18]. But, it is less than studies conducted in Russia, Bangladesh, Jordan, Nepal, north India, and Nigeria [29, 40, 42, 44, 45, 47] and it is greater than from a study conducted in Sudan [19].

The mean spleen volume was 109.34 cm^3^ which is less than studies conducted in Saud Arabia, Russia, Jordan and Nepal [29, 40, 46, 48, 49] and greater than from the studies conducted in Sudan and Ethiopia [18, 19].

The overall dimensional difference of the current study from other studies were probably due to age group difference, geographical differences, nutritional status, physical exercise, and race difference which were stated in different literature [21, 39, 47, 50–52].

The mean spleen length was lower among females than males. This is consistent with the studies conducted in Turkey, Saudi, Nigeria and Sudan [28, 39, 48, 53, 54]. This is due to fewer red cell mass in female and other genetic factors [20, 55]. But, inconsistent with study conducted in Egypt. The difference probably due to nutritional status, where women’s gain excess weight than men due to fertility consideration in case of Egypt [56, 57].

As age increase the mean spleen length, width, thickness and volume were decreased. This result is consistent with the studies conducted in Iraq, Nepal, and India [21, 35, 36, 58, 59]. This is may be due to the decrease of the number and size of B cell follicles of the white pulp of the spleen which decreases with a decrease of germinal center of spleen in older age groups [60–62]. But, inconsistent from the studies conducted in Pakistan, Jordan, and Nigeria [28, 29, 45, 63]. The difference is probably due to nutritional status where larger anthropometric measurements and obesity were observed in the studies of Pakistan, Jordan and Nigeria.

As height increase the mean spleen width and volume also increased. This is consistent with the studies conducted in Jordan, USA, India, and Sudan [19, 20, 29, 44]. This is the fact that as body parameters like height increase, the blood volume also increase that leads to large spleens for filtration. But, different from the studies conducted in Turkey, Nigeria and Egypt [43, 45, 56, 63]. This may be due to nutritional status where most of the study participants in the studies of Turkey, Nigeria and Egypt were overweight and obese than ours.

## Conclusion

The mean value of spleen dimensions for the Arba Minch town community was 10.24 cm, 4.79 cm, 3.93 cm, and 109.34 cm^3^, in length, width, thickness, and volume respectively. The mean spleen length, width, thickness and volumes among males were 10.64 cm, 4.92 cm, 4.05 cm and 119.81 cm^3^ and among females were 9.75 cm, 4.63 cm, 3.78 cm and 96.50 cm^3^ respectively. The study indicated that there is a significant morphometric difference in splenic dimensions between males and females. The mean splenic length was determined by age & sex. The mean spleen width and volume were determined by age & height and the mean Spleen thickness was determined by age only.

### Limitation of the study

Despite training on spleen dimensions measurement and repeated measurement were considered it is subjected to intra & inter observer bias. Due to resource limitation presence of illness not exclude using laboratory finding and physical activities not considered in this study. The absence of gold standard (CT and MRI) for the sonographic measurement is the other limitation of the study.

## Data Availability

All relevant data are included in the article. The dataset of this study are available from the corresponding authors upon reasonable request.

## Abbreviations

AOR: Adjusted odd ratio
AP: Antero-posterior
BMI: Body Mass Index
BSA: Body-surface area
CI: Confidence interval
CSA: Central statistical agency
CT scans: Computed tomography scans
IM: Infectious mononucleosis
MHz: Megahertz
MRI: Magnetic resonance imaging
*p*: *p* Value
PAH: Pulmonary arterial hypertension
R: Pearson correlation coefficient
RT: Radiologic technologists
SD: Standard deviation
SNNP: Southern Nations, Nationalities and peoples
SPSS: Statistical Package for the Social Sciences.
US: Ultrasonography
USA: United States of America
